# Influence of Landscape Patterns on Exposure to Lassa Fever Virus, Guinea

**DOI:** 10.3201/eid2902.212525

**Published:** 2023-02

**Authors:** Stephanie Longet, Cristina Leggio, Joseph Akoi Bore, Stephanie Key, Tom Tipton, Yper Hall, Fara Raymond Koundouno, Hilary Bower, Tapan Bhattacharyya, N’Faly Magassouba, Stephan Günther, Ana-Maria Henao-Restrapo, Jeremy S. Rossman, Mandy Kader Konde, Kimberly Fornace, Miles W. Carroll

**Affiliations:** Pandemic Sciences Institute, Oxford, UK (S. Longet, J.A. Bore, T. Tipton, M.W. Carroll);; UK Health Security Agency, Porton Down–Salisbury, UK (C. Leggio, Y. Hall, M.W. Carroll);; London School of Hygiene and Tropical Medicine, London, UK (C. Leggio, S. Key, H. Bower, T. Bhattacharyya, K. Fornace);; Ministry of Health, Conakry, Guinea (J.A. Bore, F.R. Koundouno);; Center of Excellence for Training, Research on Malaria & Priority Diseases in Guinea, Conakry (J.A. Bore, M.K. Konde);; University of Kent School of Biosciences, Canterbury, UK (J.A. Bore, J.S. Rossman);; Projet Laboratoire Fièvres Hémorragiques, Conakry (N. Magassouba);; Bernhard Nocht Institute for Tropical Medicine, Hamburg, Germany (S. Günther);; German Center for Infection Research, Hamburg–Lübeck–Borstel–Riems, Germany (S. Günther);; World Health Organization, Geneva, Switzerland (A.-M. Henao-Restrapo);; Research-Aid Networks, Chicago, Illinois, USA (J.S. Rossman);; University of Glasgow, Glasgow, Scotland, UK (K. Fornace)

**Keywords:** Lassa virus, seroepidemiology, IgG responses, fragmentation, land use land cover change, Guinea, viruses

## Abstract

Lassa fever virus (LASV) is the causative agent of Lassa fever, a disease endemic in West Africa. Exploring the relationships between environmental factors and LASV transmission across ecologically diverse regions can provide crucial information for the design of appropriate interventions and disease monitoring. We investigated LASV exposure in 2 ecologically diverse regions of Guinea. Our results showed that exposure to LASV was heterogenous between and within sites. LASV IgG seropositivity was 11.9% (95% CI 9.7%–14.5%) in a coastal study site in Basse-Guinée, but it was 59.6% (95% CI 55.5%–63.5%) in a forested study site located in Guinée Forestière. Seropositivity increased with age in the coastal site. We also found significant associations between exposure risk for LASV and landscape fragmentation in coastal and forested regions. Our study highlights the potential link between environmental change and LASV emergence and the urgent need for research on land management practices that reduce disease risks.

Lassa virus (LASV), the cause of Lassa fever in humans, is an enveloped and negative-sense, single-stranded RNA virus belonging to the *Arenaviridae* family (*1*). Initial clinical manifestations, such as fever, weakness, headache, respiratory and gastrointestinal symptoms, and conjunctivitis, are often nonspecific and could indicate of several other febrile illnesses (*2*). The mortality rate is unclear because of inconsistent reporting practices and lack of diagnostic services in most endemic areas but is estimated to be 5%–20% in hospitalized patients (*2*,*3*).

The Natal multimammate rat (*Mastomys natalensis*) is considered the main natural reservoir of LASV. It is a commensal rodent and agricultural pest that aggregates in human dwellings and surrounding fields (*4*). Both zoonotic and nonzoonotic transmission mechanisms have been described (*5*). Human LASV infections most commonly occur through infected rodent excreta, contaminated food, and inhalation of aerosols from rodent urine or droppings (*6*,*7*). Person-to-person transmission may also occur through exposure to contaminated bodily fluids (*5*,*8*).

The distribution of the Natal multimammate rat is a major risk factor for LASV exposure. The presence of the rat increases in houses during the dry season because of restricted food supply, which enhances the risk for humans encountering rodents or being in contact with excreta (*9*). Climatic variability leading to changes in human agriculture, food storage practices, and land cover can affect rodent population dynamics (*10*). Recent studies have also identified additional rodent reservoirs (e.g., African wood mouse and Guinea multimammate mouse), indicating the potential for a wider ecologic niche for the virus (*11*,*12*).

LASV is endemic in West Africa, particularly in Nigeria, Sierra Leone, Liberia, and Guinea (*13*,*14*). Estimates on annual human cases of LASV infections in West Africa range from 100,000 to 300,000, with 5,000–10,000 estimated deaths per year and 80% asymptomatic infections. Further epidemiologic studies are needed because these figures have been extrapolated from 1 longitudinal study conducted in Sierra Leone in 1987 (*15*). Characterizing the distribution and transmission intensity of LASV in endemic areas is essential for designing effective surveillance and disease prevention and control programs.

Exposure to LASV may lead to IgG seroconversion (*16*). Analysis of age-stratified serologic responses is a powerful tool to estimate exposure to LASV and to reconstruct historical patterns of transmission and distribution of previously unreported cases (*17**–**19*). Measurement of LASV antibodies has been used to detect circulation of LASV across Guinea in previous studies conducted in the 1990s and early 2000s (*6*,*20*,*21*). However, the current incidence and distribution of LASV in Guinea is largely unknown.

Our study aimed to characterize LASV exposure in human populations in 2 ecologically diverse regions of Guinea: the Basse-Guinée (coastal site); and the Guinée Forestière region, which includes the town of Guéckédou and Macenta Prefecture (forested site). We describe LASV-specific IgG levels within these populations, estimate transmission intensity by using age-stratified antibody responses, and explore associations with demographic and environmental risk factors, including fragmentation (the breaking apart of an organism’s preferred habitat into smaller patches).

## Materials and Methods

### Ethics Considerations

The part of the study conducted in Basse-Guinée (coastal site) was approved by the London School of Hygiene and Tropical Medicine Ethics Committee (reference no. 17429) and the Guinean Ministry of Health. For the part conducted in the town of Guéckédou and Macenta Prefecture (forested site), ethics approval for research projects on human volunteers was obtained from the Republic of Guinea’s National Ethics Committee for Health Research on March 16 (approval no. 012/CNERS/17).

### Study Sites

**Figure 1:**
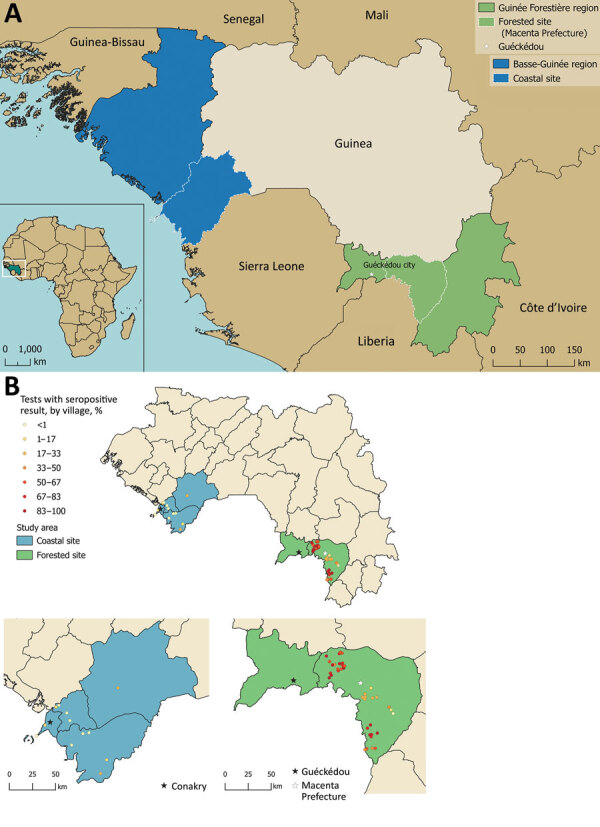
Study sites and Lassa fever virus (LASV) seropositivity for study of influence of landscape patterns on exposure to LASV, Guinea. A) Map of West Africa and Guinea showing coastal and forested sites for the study. Inset shows location of Guinea in Africa. B) LASV-specific IgG seropositivity (%) by village across the coastal and forested sites.

Guinea is administratively classified into regions on the basis of broad ecologic characteristics, including the Basse-Guinée and Guinée Forestière regions (*22*). The samples used in this study were collected during 3 separate studies in 2 geographically diverse regions of Guinea: Basse-Guinée (coastal site); and Guinée Forestière, including Macenta Prefecture and the town of Guéckédou (forested site) (*22*). Basse-Guinée (population 2,749,909, area 60,059 km^2^) is the coastal region of Guinea, extending for 30–40 miles inland from the Atlantic Ocean to the coastal plains. Basse-Guinée is mainly covered by mangroves and cultivated areas. The rainy season lasts 3 months per year in this region (Figure 1, panel A). Macenta Prefecture (population 297,779, area 8,600 km^2^) and the town of Guéckédou (population 346,908) are located to the southeast of the country, close to the border with Liberia and Sierra Leone, within Guinée Forestière. In addition to primary and secondary forests (umbrella and palm trees), which mainly cover this area, mountains and savannah can also be found. The rainy season lasts 9 months per year in this region (*23**–**25*) (Figure 1, panel A).

### Study Participants

We obtained written informed consent from participants. Consent forms stated that blood samples would be tested for antibodies specific to Ebola virus and other pathogens, including LASV.

Samples from Basse-Guinée were collected in 2016 as part of a clinical trial evaluating the safety and immunogenicity of the vesicular stomatitis virus–based Ebola virus vaccine in contacts of Ebola survivors. That trial was performed in partnership with Guinea’s Ministry of Health, the World Health Organization, and Public Health England. For this study site, we randomly selected 702 samples taken before vaccination and tested them for the presence of LASV-specific IgG. Data recorded included the person’s age, sex, and village of residence at the time of vaccination. The selected samples originated from 7 prefectures in Basse-Guinée, encompassing 24 subprefectures and 29 villages or neighborhoods.

We collected samples from Macenta Prefecture during February–December 2017 as part of a seroepidemiologic study on Ebola virus that included both affected and nonaffected villages during the 2013–2016 Ebola virus disease (EVD) outbreak (*26*). In total, 517 participants living in 7 subprefectures encompassing 44 villages were enrolled. Volunteers were recruited upon agreement. The volunteers were from the general population of the village, including bush meat hunters, their family members, local healthcare workers. Volunteers <16 years of age, pregnant persons, travelers or visitors, and persons with chronic disease were excluded from the original study. In total, we tested 516 samples from the Macenta Prefecture cohort for the presence of LASV-specific IgG. One sample was not analyzed because not enough serum remained to run the assay. For our analysis, we included 68 samples from Guéckédou, collected in 2018 in the context of a longitudinal study of EVD survivors, in the Macenta Prefecture population because both Guéckédou and Macenta Prefecture are located within Guinée Forestière.

### Blood Sample Collection and Processing

We collected samples (5–20 mL) of human peripheral blood into nonanticoagulant Vacutainer blood collection tubes (Becton, Dickinson and Company, https://www.bd.com). Blood clotted within 2 hours, and we transported samples to the laboratory at 4°C. We centrifuged samples at 2,000 × *g* for 10 min at room temperature and aliquoted serum into 2-mL microtube tubes (Starsted, https://www.sarstedt.com) in an air-purifying class II microbiologic safety cabinet (Envair, https://envairtechnology.com). We shipped aliquots of serum samples at –20°C; we processed and stored samples at –80°C at Public Health England’s Porton Down laboratories.

### Samples Analysis by LASV-Specific ELISA

LASV exposure, which is defined as the contact with the virus, can be assessed by the presence of LASV-specific antibodies in the blood (*27*). We used the Blackbox LASV IgG Kit from Bernhard Nocht Institute for Tropical Medicine in Germany (https://www.bnitm.de) for qualitative serologic detection of acute or past LASV infection (*28*,*29*). We used the kit according to the manufacturer’s instructions; all reagents were provided, including positive and negative controls. We ran each sample as single replicate. After washing them with a saline solution, we transferred 25 μL of diluted biotinylated recombinant LASV nucleoprotein onto the ELISA plate and added 25 μL of diluted sample before plate incubation for 24 h at 4°C. After a wash step, we added 50 μL of diluted streptavidin horseradish peroxidase and incubated the plate for 1 h at 4°C. Finally, we developed the plate by using 100 μL/well of 3,3′,5,5′-tetramethylbenzidine peroxidase substrate and stopped the chemical reaction by using 100 μL/well of 1 mol/L H_2_SO_4_. We obtained optical density (OD) readings at wavelengths 450–620 nm (OD_450–620_) by using SpectraMax plate reader with SoftMaxPro 9 software (Molecular Devices, https://www.moleculardevices.com). OD is a term used to describe the propagation of the light induced by the reaction. We interpreted results as valid if the following criteria were met: OD_450–620_ of negative control <0.10 and OD_450–620_ of positive control >1.00. We used average absolute OD_450–620_ absorbance value for negative control samples OD_Neg,AV_ to calculate the assay cutoff OD_CO_ according to the formula OD_Neg,AV +_ 0.150 = OD_CO_. Index values (IVs) are values comparing the ODs of the sample and the cutoff. We calculated IV for the tested samples (IV_sample_) by using the formula IV_sample_ = OD_450–620_ (sample)/OD_CO_. Results classification for IV was carried out as follows: IV_sample_ = >1.00 = positive; 0.90 <IV_sample_ <1.00 = equivocal; IV_sample_ <0.90 = negative.

### Environmental and Spatial Covariates

To assess the effect of land cover and other environmental covariates on LASV exposure, we assembled a dataset of remote sensing–derived land cover maps from 2017 at 100 m resolution (*30*). We reclassified land cover into the following standardized categories, which are mutually exclusive: closed forest (canopy cover >70% of surface area), open forest (canopy cover 15%–70% of surface area), shrubs (woody perennial plants with persistent stems <5 m), vegetation (plants without persistent stems and canopy cover <10% of surface area), and urban or built up areas.

Village populations had been recorded during field surveys. Because villages were only geolocated to centroids, we extracted environmental data within buffer radii of 500 m, 1 km, 2 km, 5 km, and 10 km from the village centroid to represent the possible distributions of the village and lands used (*31*). For all buffer radii, we extracted the proportions of different land cover types and the mean canopy cover. To evaluate the importance of landscape configuration (spatial arrangement of different elements of the landscape) (*32*), we also extracted class-level metrics, including perimeter area ratio, shape index (patch perimeter divided by the minimum possible patch perimeter), and fractal dimension index (an index of shape complexity) (*33*). Those indices are commonly used as measures of landscape fragmentation, with higher values indicating greater levels of fragmentation (*34*,*35*).

### Models and Statistical Analyses

Because assays were not validated for quantitative antibody responses, we fit all models by using classified values of seropositivity. To characterize transmission intensity in both sites, we integrated data on age-specific frequency of seropositivity. We fit reversible catalytic models to age-specific seroprevalence data by using maximum-likelihood methods (*17*). Those models generate age-specific seroprevalence curves, enabling the calculation of a seroconversion rate (SCR), which representing the force of infection for the community, a measure of transmission intensity (number of infections per person per year). SCR is the rate at which seronegative persons become seropositive; although it is a measure of transmission intensity, SCR is not incidence because it does not capture seropositive persons who are repeatedly exposed. Those models are widely used to characterize transmission of endemic diseases such as malaria (*36*). We used profile likelihood plots to explore possible historical changes in transmission intensity represented by the SCR and selected final models by using likelihood ratio tests (*37*).

We additionally assessed whether fine-scale patterns of seroconversion were associated with landscape characteristics. Because environmental variables can be associated with risk at different spatial scales, we used a data-driven approach to identify environmental risk factors (*38*). Because our environmental dataset was high-dimensional and highly correlated, we first performed mixed-effects logistic regression models for each environmental variable at each spatial scale (126 in total). We excluded from subsequent analysis any environmental variables with p values >0.2. We used Pearson correlation to identify highly correlated variables (Pearson correlation coefficient >0.75). For these highly correlated variables (e.g., environmental factors at different spatial scales), we identified a single variable for inclusion based on the Akaike information criteria. Because sampling frames were different between sites, we ran models separately for both sites, using the primary outcome as the binary classification for person-level seropositivity. We fitted models with village as a random effect to account for observations within villages lacking independence. We first assessed associations with key demographic factors, including sex, continuous age, and age category. For the forested site model, we included only environmental predictors because demographic factors (age and sex) were not associated with exposure risks. For the coastal site, we included age category in all models in addition to environmental predictors. When assessing for inclusion in the multivariate analysis, we considered univariate results to be statistically significant at p<0.2 to avoid exclusion of variables that alone lack significance but contribute in the presence of other variables. We conducted final multivariate model selection by using the dredge function in the MuMIn package (*39*), selecting the best model according to the Akaike information criteria and using model averaging approaches to obtain average regression coefficients for the best fitting models. The code for environmental data extraction and model fitting is available online (https://github.com/kfornace/LFV_environmental).

## Results

In this study, we assessed LASV IgG seropositivity in 1,286 persons by using serum samples collected in 2 sites in Guinea during 2016–2018. In the coastal site, we analyzed 702 samples (Table; Figure 1, panel A; Appendix Table 1). In the forested site, we analyzed 516 samples from Macenta Prefecture and 68 from the town of Guéckédou (Table; Figure 1, panel A; Appendix Table 2).

**Table:** Characteristics of Lassa fever study participants from coastal and forested sites, Guinea

Characteristic	Coastal site	Forested site
No. participants	702	584*
Sex, no. (%)		
M	459 (65.4)	322 (51.1)
F	243 (34.6)	262 (44.9)
Mean age (range), y	32.3 (6–99)	38.8 (17–90)
Sampling dates	2016	2017–2018

*516 in Macenta Prefecture and 68 in the town of Guéckédou.

We observed that exposure to LASV was highly heterogenous both between and within each study site (Figure 1, panel B). The LASV IgG seropositivity was 11.9% (95% CI 9.7%–14.5%) in the coastal site (Figure 1, panel B; Appendix Table 1), whereas it was 59.6% (95% CI 55.5%–63.5%) in the forested site (Figure 1, panel B; Appendix Table 2).

Demographic characteristics of exposed persons varied by study site. In the coastal site, seropositivity in women was 9.0% (95% CI 5.8%–13.1%) and in men was 13.5% (95% CI 11.0%–17.0%). For the forested site, seropositivity in women was 61.8% (95% CI 56.0%–67.7%) and in men was 58.0% (95% CI 52.5%–63.3%). Sex was not significantly associated with seropositivity in either site (coastal site, odds ratio 1.25 [95% CI 0.69–2.27], p = 0.46; forested site, odds ratio 0.90 [95% CI 0.61–1.33], p = 0.59).

**Figure 2:**
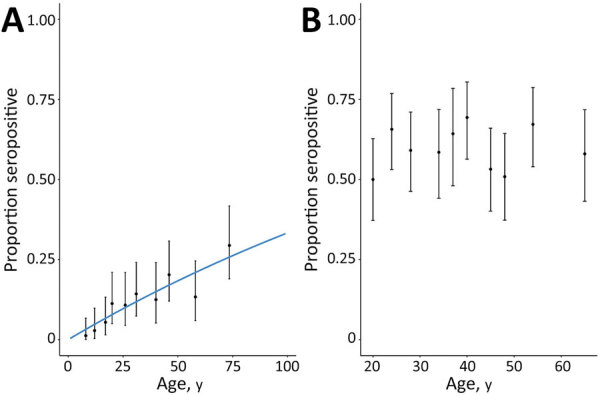
Lassa fever virus (LASV) seropositivity by age for study of influence of landscape patterns on exposure to LASV, by study site, Guinea. Dots show the proportion of LASV seropositive persons (no. positive/total no.) by age decile for the coastal site (A) and the forested site (B). Blue line indicates modeled seroconversion rate curve. Error bars indicate 95% CIs.

Age patterns of seropositivity differed markedly between sites. In the coastal site, exposure was strongly associated with age, and seropositivity increased with age (Figure 2, panel A). In that population, reversible catalytic models estimated the force of infection (SCR) as 0.0041 (95% CI 0.0033–0.0050). We observed no changes in historical transmission intensity (p = 0.328). In contrast, in the forested site, we identified no clear age patterns, and neither continuous nor categorical age were associated with person-level seropositivity (Figure 2, panel B). Although age ranges for included persons were different between both sites (Table), we detected substantial differences in exposure risks in similar age groups between sites. For example, persons 17–20 years of age had an estimated seropositivity of 5.4% (95% CI 1.5%–13.3%) in the coastal site, compared with 50.0% in the forested site (95% CI 37.2%–62.8%). The high proportions of seropositivity in the youngest age groups and lack of association with age precluded fitting reversible catalytic models for the forested region.

**Figure 3:**
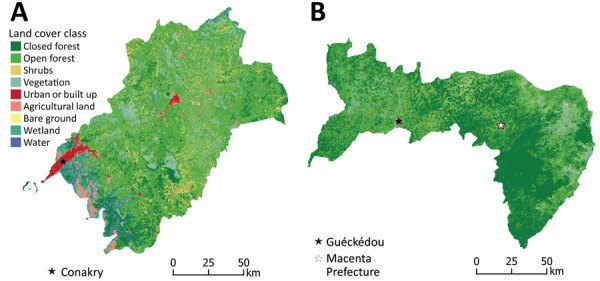
Land cover maps of coastal (A) and forested (B) sites for study of influence of landscape patterns on exposure Lassa fever virus, Guinea.

**Figure 4:**
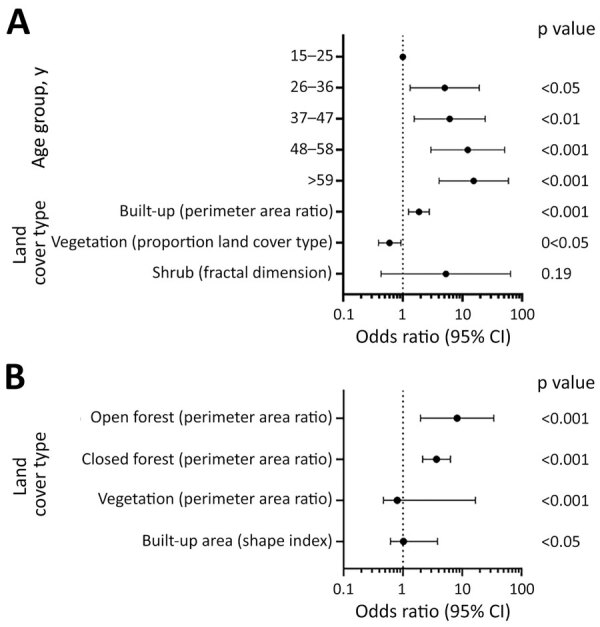
Associations between Lassa fever virus exposure risk and environmental factors by study site for study of influence of landscape patterns on exposure Lassa fever virus, Guinea. A) Coastal site. B) Forested site.

Despite clear differences in seropositivity between sites, we identified strong associations between exposure and environmental factors for both sites (Figure 3). As characterized by perimeter area ratios, fragmentation was strongly positively associated with exposure risk for both sites (Figure 4; Appendix Tables 3, 4). In the coastal site, odds of exposure increased with the perimeter area ratio of built-up areas but were lower where the proportion of vegetation in the landscape was higher. The fractal dimension index measuring fragmentation of shrubland was not significantly associated with exposure risk but improved overall model fit (Figure 4, panel A; Appendix Table 3). In the forested site, higher perimeter area ratios of both open and closed forests and the shape index of built areas, all indicating higher fragmentation levels, were positively associated with risk, whereas for vegetation a high perimeter area ratio was protective (Figure 4, panel B; Appendix Table 4). Across both sites, significant variables were identified at different spatial scales.

## Discussion

This study aimed to estimate LASV transmission intensity from serologic data in Guinea and analyze environmental risk factors. We found that LASV seropositivity was significantly greater in the forested site than in the coastal site. In 1993, Lukashevich et al. (*20*) also found high LASV nucleoprotein–specific IgG positivity among inhabitants of tropical forest (Guéckédou, Yomou, and Lola prefectures) and Guinea savannah (Faranah and Kindia prefectures) (25%–55%) and a lower positivity among inhabitants of mountainous areas (Pita, Labe, and Mali prefectures) and coastal areas (Boffa and Boké prefectures) (4%–7%).

We found that sex was not significantly associated with seropositivity in either site. This result was consistent with previous studies conducted in Guinea and Sierra Leone (*15*,*20*).

Our findings showed a significant increase in LASV IgG seropositivity with age in the coastal site but not in the forested site. Because no persons <16 years of age were included in our study, additional serologic studies of children would be needed to assess the age of exposure and how this is affected by environment. Although previous studies did not detect any correlation between antibody prevalence and age in these regions (*20*), differences in age patterns of seropositivity between regions were observed in other studies in West Africa. A study conducted in a forested region in 2000 found that children <10 and adults 20–29 years of age had the highest LASV IgG prevalence (*6*). A study conducted in Sierra Leone found that antibody prevalence increased with age, reaching a peak at 20–50 years of age and decreasing thereafter (*15*). A similar trend was reported in a 2015 study conducted in Nigeria (*40*). Age, often associated with differences in occupation or daily activities, may play a role in exposure (e.g., bushmeat hunters are mainly young to middle-age men and may have more contact with rodents). However, our results in the forested site suggest that in some areas, age-independent factors such as environmental features may have a greater effect on exposure risk.

All models showed an association between landscape patterns and exposure risk for both study sites (Appendix Tables 3–6). Associations between landscape fragmentation and exposure risk to zoonotic diseases also have been observed for malaria (*41*,*42*) and Ebola virus (*43*). Fragmentation may affect disease transmission by increasing spatial overlap between humans and wildlife populations at habitat edges (*41*). In our study, the fragmentation and the proportion of forests appear to affect exposure risk but their exact role in the complex dynamics of LASV circulation remains to be determined. Because the distribution of Natal multimammate rats is the leading LASV risk factor (*9*), the link between landscape patterns and the distribution of rodent reservoirs should be further explored. Of interest, one study found that the proportion of LASV-infected Natal multimammate rats was higher in forested regions than in coastal regions (*13*).

One limitation of our study is that, although the Blackbox IgG ELISA was shown to be sensitive (90%) and very specific (99%) (*28*), it was not tested against other arenaviruses because of a lack of suitable serum panels. We cannot fully exclude the potential circulation of related or yet undescribed arenaviruses in specific regions (*44*) and a partial cross-reactivity of antibodies against other arenaviruses. In addition, because the samples were collected as part of a vaccine evaluation and a seroepidemiologic study on Ebola virus, the sampling procedure was not standardized between the studies. Because the sampling approaches were not designed to be representative of the wider populations across Guinea, the estimates cannot be used to generalize about population-level disease risks.

In addition, behavioral exposure risks (e.g., occupation, medical history, hunting or foraging habits, location of previous residence, methods for food storage, and rodent trapping around the house) could not be evaluated because we did not collect person-level questionnaire data. Future studies could assess the number of persons in the household, the quality of the building, and proximity to rodent burrows. This study measured only IgG in serum samples collected at a single timepoint. As such, our data do not enable us to capture the potential effect of changes in seasonal rodent activity, vegetation patterns surrounding the villages, or the fluctuation of LASV persistence in rodents linked to climate variation. Because persons tend to move frequently across wide regions, either to find work during harvest season or to hunt (*45*) or in response to EVD hotspots, they may have been exposed to LASV in a different location from their current residence.

Because samples were not collected uniformly from younger age groups and a high proportion of persons from the forested region were exposed by 18 years of age, transmission intensity could not be directly compared between sites using reversible catalytic models. Although population-based epidemiologic studies are required to characterize wider LASV exposure and risk factors, the differences between these populations surveyed highlight the potential heterogeneity of LASV transmission intensity in Guinea.

In conclusion, LASV dynamics result from complex interactions between several human and environmental factors. Our study compared the LASV-specific IgG seropositivity in coastal and forested regions in Guinea and described associations between exposure to LASV and landscape patterns. Enhanced knowledge of regions with a potentially increased risk for LASV exposure can help improve epidemiologic surveillance; devise a global approach to optimize the health of humans, animals, and the environment; and highlight potential trial sites to assess new LASV vaccines and therapeutics.

Appendix Additional information about influence of landscape patterns on exposure to Lassa fever virus, Guinea.
