# Supplementary material for: Influence of Landscape Patterns on Exposure to Lassa Fever Virus, Guinea
Source: Emerg Infect Dis. 2023 Feb;29(2):304–13. doi: 10.3201/eid2902.212525 (PMC9881776; doi:10.3201/eid2902.212525)
Supplement: Appendix — Additional information about influence of landscape patterns on exposure to Lassa fever virus, Guinea. [file 21-2525-Techapp-s1.pdf]

Article DOI: https://doi.org/10.3201/eid2902.212525

Influence of Landscape Patterns on Exposure to Lassa Fever Virus, Guinea

Appendix

Appendix Table 1. LASV IgG seropositivity in the coastal site shown by prefectures

Prefectures	No. Samples	No. Positives	Seropositivity (95% CI)
Boke	40	5	12.5 (2.3–22.8)
Conakry	100	2	2.0 (0–4.7)
Coyah	27	0	0
Dubreka	84	2	2.4 (0–5.6)
Forecariah	406	67	16.5 (12.9–20.1)
Fria	10	1	10.0 (0–28.6)
Kindia	35	7	20.0 (6.8–33.3)

Appendix Table 2. LASV IgG seropositivity in the forested site (Macenta Prefecture/town of Guéckédou) shown by subprefectures

Prefectures	Subprefectures	No. Samples	No. Positives	Seropositivity (95% CI)
Macenta	Binikala	48	27	56.25 (41.18–70.52)
Macenta	Bofossou	190	150	78.95 (72.46–84.51)
Macenta	Coyamah	42	21	50.00 (34.19–65.81)
Macenta	Fassankoni	76	66	86.84 (77.13–93–51)
Macenta	Oremai	76	16	21.05 (12.54–31.92)
Macenta	Seredou	71	12	16.90 (9.05–27.66)
Macenta	Wattanka	13	10	76.92 (46.19–94.96)
Guéckédou	Guéckédou (town)	68	46	67.65 (55.21–78.49)

Appendix Table 3. Associations between exposure risk and environmental factors in the coastal site determined by the best fitting model

Variable			OR	95% CI	p value
Age group, y	Positive tests/tests taken per age category 84/702				
15–25	3/152		Baseline		
26–36	15/171		5.02878498	(1.32–19.13)	<0.05
37–47	15/121		6.10695218	(1.55–24.12)	<0.01
48–58	12/99		12.2878142	(2.95–51.1)	<0.001
≥59	13/64		15.4292165	(4.04–58.95)	<0.001
Land cover type	Metric	Scale	OR	95% CI	p value
Built-up	Perimeter area ratio	2000	1.86180536	(1.24–2.79)	<0.001
Vegetation	Proportion land cover type	10000	0.59804749	(0.39–0.93)	<0.05
Shrub	Fractal dimension	5000	5.27593943	(0.43–64.19)	0.19

Appendix Table 4. Associations between exposure risk and environmental factors in the forested site determined by the best fitting model

Land cover type	Metric	Scale, m	OR	95% CI	p value
Open forest	Perimeter area ratio	20000	1.980333	(8.22–34.19)	<0.001
Closed forest	Perimeter area ratio	10000	3.683982	(2.15–6.32)	<0.001
Vegetation	Perimeter area ratio	20000	16.762015	(0.47–0.80)	<0.001
Built-up area	Shape index	5000	0.613116	(1.02–3.83)	<0.05

Appendix Table 5. Associations between exposure risk and environmental factors in the coastal site determined by the conditional model-averaged coefficients

Age group, y			Estimate	SE	p value
15–25			1.27	0.68	0.06
26–36			1.62	0.68	<0.05
37–47			1.81	0.70	<0.05
48–58			2.50	0.73	<0.001
≥59			2.73	0.69	<0.001
**Land cover type**	**Metric**	**Scale, m**	**Estimate**	**SE**	**p value**
Built-up area	Perimeter-area ratio	2000	0.58	0.21	<0.001
Shrub	Fractal dimension	5000	2.44	2.41	0.31
Vegetation	Proportion of land cover type	10000	−0.46	0.25	0.07
Built-up area	Shape index	5000	−0.54	0.37	0.15
Built-up area	Perimeter-area ratio	5000	0.28	0.24	0.24
Built-up area	Proportion of land cover type	10000	−0.16	0.21	0.45
Vegetation	Shape index	1000	−0.24	0.32	0.44
Closed forest	Perimeter-area ratio	5000	−0.13	0.26	0.62
Open forest	Shape index	500	0.17	0.28	0.55
Closed forest	Perimeter-area ratio	500	0.10	0.18	0.57
Vegetation	Shape index	500	−0.09	0.21	0.66
Vegetation	Fractal dimension	1000	−0.11	0.37	0.76

Appendix Table 6. Associations between exposure risk and environmental factors in the forested site determined by the conditional model-averaged coefficients

Land cover type	Metric	Scale, m	Estimate	SE	p value
Built-up area	Shape index	5000	0.7084	0.3923	0.07
Closed forest	Perimeter-area ratio	10000	1.1895	0.3468	<0.001
Open forest	Perimeter-area ratio	20000	2.5861	0.496	<0.001
Vegetation	Perimeter-area ratio	20000	−0.5491	0.157	<0.001
Built-up area	Shape index	20000	0.4591	0.2889	0.11
Shrub	Perimeter-area ratio	5000	−0.2176	0.1743	0.21
Built-up area	Perimeter-area ratio	5000	0.1387	0.149	0.35
Open forest	Fractal dimension	5000	0.4373	0.4261	0.31
Shrub	Perimeter-area ratio	2000	−0.1373	0.1965	0.49
Built-up area	Shape index	2000	−0.1261	0.2938	0.67
Closed forest	Perimeter-area ratio	500	−0.2071	0.2672	0.44
Open forest	Fractal dimension	2000	0.7379	2.0064	0.71
